# Low-Molecular-Weight Polyethyleneimine Grafted Polythiophene for Efficient siRNA Delivery

**DOI:** 10.1155/2015/406389

**Published:** 2015-10-11

**Authors:** Pan He, Kyoji Hagiwara, Hui Chong, Hsiao-hua Yu, Yoshihiro Ito

**Affiliations:** ^1^Nano Medical Engineering Laboratory, RIKEN, 2-1 Hirosawa, Wako, Saitama 351-0198, Japan; ^2^Responsive Organic Materials Laboratory, RIKEN, 2-1 Hirosawa, Wako, Saitama 351-0198, Japan; ^3^Institute of Chemistry, Academia Sinica, 128 Academic Road, Section 2, Nankang, Taipei 11529, Taiwan

## Abstract

Owing to its hydrophilicity, negative charge, small size, and labile degradation by endogenous nucleases, small interfering RNA (siRNA) delivery must be achieved by a carrier system. In this study, cationic copolymers composed of low-molecular-weight polyethylenimine and polythiophenes were synthesized and evaluated as novel self-tracking siRNA delivery vectors. The concept underlying the design of these copolymers is that hydrophobicity and rigidity of polythiophenes should enhance the transport of siRNA across the cell membrane and endosomal membrane. A gel retardation assay showed that the nanosized complexes formed between the copolymers and siRNA were stable even at a molar ratio of 1 : 2. The high cellular uptake (>80%) and localization of the copolymer vectors inside the cells were easily analyzed by tracking the fluorescence of polythiophene using fluorescent microscopy and cytometry. An *in vitro* luciferase knockdown (KD) assay in A549-luc cells demonstrated that the siRNA complexes with more hydrophobic copolymers achieved a higher KD efficiency of 52.8% without notable cytotoxicity, indicating protein-specific KD activity rather than solely the cytotoxicity of the materials. Our polythiophene copolymers should serve as novel, efficient, low cell toxicity, and label-free siRNA delivery systems.

## 1. Introduction

Since the pioneering work of RNA interference in 1998, gene suppression using small interfering RNA (siRNA) has received significant attention recently as an approach for treating inherited or acquired diseases [1]. However, finding an efficient drug delivery system (DDS) remains a major challenge for translating siRNA to the clinic [2]. Naked siRNA as unprotected oligonucleotides have a very short half-life* in vivo* (seconds to minutes) as a result of degradation by endogenous nucleases and rapid kidney filtration from circulation owing to their small size [3]. Following cellular internalization, the siRNA must also escape the endosome, because siRNA must enter the cytosol to have a therapeutic effect [4]. Thus, effective vehicles for siRNA delivery must demonstrate siRNA binding, low cytotoxicity, effective cellular uptake, and endosome escape and most importantly show evidence of siRNA-induced knockdown [5, 6].

Branched polyethylenimine (PEI) with a molecular weight of 25 kDa, named PEI-25K, and its derivatives have been the most popular cationic polymers for* in vitro* and* in vivo* gene delivery. This is because of their superior buffering capacity, which allows cargoes to escape the endosome to the cytoplasm by a hypothesized “proton sponge” mechanism [7, 8]. However, high-molecular-weight PEI is still limited by cytotoxic issues, as assessed by the* in vitro* metabolic activity of cells [9]. Recently, low-molecular-weight PEIs with better biocompatibility, but low gene loading capacity, have proven to be valuable gene vectors after hydrophobic modification or cross-linking [10, 11]. Hydrophobic alkyl-modified PEI-2k/carbon-dot nanocomposites were found to be efficient for* in vitro* gene delivery with low cytotoxicity [12]. Thus, it is likely that there will be more* in vitro* and* in vivo* studies using hydrophobic-modified low-molecular-weight PEI for gene delivery [13].

Conjugated polymers such as polythiophenes [14] and poly(p-phenylene ethynylene) [15] and their nanoparticles [16] have emerged as novel gene delivery vectors, because of their potential cell-penetrating ability owing to their rigid chains, and such polymers are easy to use as traceable delivery vehicles [17]. For example, monodispersed polyfluorene nanoparticles showed outstanding RNA-binding capacity and induced a knockdown efficiency of 23.9% with no significant cytotoxicity [18]. Jubeli et al. recently reported the potential of polyene-based cationic lipids as visually traceable siRNA transfer reagents for inhibition of luciferase expression [19]. To improve the knockdown or silencing efficiency is a challenge for conjugated polymeric gene carriers.

**Scheme 1:**
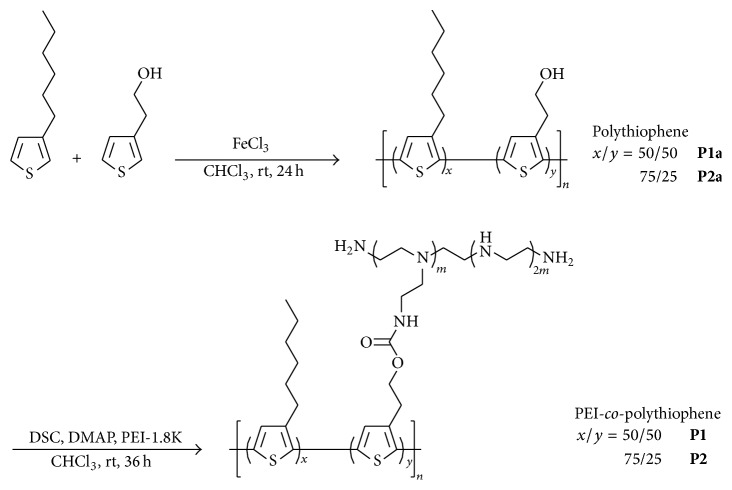
Synthesis of PEI-*co*-polythiophenes copolymers** P1** and** P2** for siRNA delivery.

In our previous study, polyethylenedioxythiophenes with a cell-membrane-mimicking strategy were synthesized and showed specific neuron targeting and enhanced neuron cell adhesion and proliferation [20]. Similar to other conjugated polymers, polythiophenes may have possible cell-membrane penetrating ability via the rigid hydrophobic main chain. In this study, we designed copolymers composed of PEI-1.8K and hydrophobic polythiophenes (as shown in Scheme 1) as high-performance siRNA carriers. PEI-1.8K acted as a gene-condensing agent to form positively charged nanosized complexes with siRNA with minimal cytotoxicity. Moreover, polythiophenes with hydrophobic hexyl groups were employed both for fluorescence label-free function and for enhancing permeation across the cell membrane. The formation of the polymer/siRNA complex, cellular uptake and localization of fluorescence polymers, and a siRNA-mediated luciferase knockdown assay were carried out to evaluate this novel visualized siRNA vector.

## 2. Materials and Methods

### 2.1. Materials for Polymer Synthesis

Branched polyethylenimine (PEI-1.8K), anhydrous ferric chloride (FeCl_3_), 4-(dimethylamino)pyridine (DMAP), and N,N′-disuccinimidyl carbonate (DSC) were purchased from Wako Pure Chemical Industries, Ltd. (Tokyo, Japan) and were used without further purification. Dialysis membranes (*M*_mwco_ = 7,000 Da) were obtained from Spectro Laboratories Inc. (Sylmar, CA, USA) and were used according to the manufacturer's instruction. 3-Hexylthiophene, 3-thiophene ethanol, and all other chemicals were from Tokyo Kasei Kogyo Co. Ltd. (TCI, Tokyo, Japan) and were used without further purification.

### 2.2. Cell Culture and siRNA

A549 cells stably expressing the luciferase gene (A549-luc) were grown in F-12K (Invitrogen, Life Technologies, Rockville, MD, USA) supplemented with 10% fetal bovine serum (FBS) and 200 *μ*g/mL geneticin (Invitrogen). The cells were maintained at 37°C in a humidified atmosphere with 5% CO_2_. All gene knockdown assays were performed by the siRNA against luciferase gene (sense strand: 5′-cuuAcGcuGaGuAcuucGAT^*∗*^T-3′ and antisense strand: 5′-UCGAAGUACUCAGCGUAAGT^*∗*^T-3′ [4]) and mismatch control siRNA, which was confirmed to have no significant knockdown activity against luciferase gene.

### 2.3. Synthesis of Copolymers

#### 2.3.1. Synthesis of Poly(3-hexylthiophene-*co*-thiopheneethanol)** P1a** and** P2a**

**P1a** and** P2a** were synthesized by a previously reported, anhydrous FeCl_3_ catalyzed, chemical oxidative coupling approach with different feed ratios of the thiophene monomers [21]. A mixture of monomer solutions in CHCl_3_ was added to the suspension of anhydrous FeCl_3_ (4 equal moles of monomers) in CHCl_3_ under nitrogen and then stirred for 24 h at room temperature. After dedoping by ammonia water, the resulting fluorescent polythiophenes were purified by dissolving in CHCl_3_, precipitating in methanol, and then drying. The obtained** P1a** and** P2a** are dark red solids.

#### 2.3.2. Conjugation of PEI-1.8K

**P1a** was dissolved in dry chloroform (1 mM pendent hydroxyl group) and then DMAP (5 mM) and DSC (5 mM) were added slowly under magnetic stirring. After 6 h, a chloroform solution of PEI-1.8K (1 mM) was added to the reaction mixture and stirred for another 36 h [22]. The reaction mixture was concentrated and precipitated in hexane to remove overdosed DMAP and DSC. Excess PEI was removed by dialysis in methanol and then water for 3 days. The final copolymers were obtained as rust red solids after freeze-drying.

#### 2.3.3. Characterization of Polymers

The structures of the synthesized compounds were identified by ^1^H NMR spectroscopy (JEOL AL400, Tokyo, Japan). The weight-average molecular weight (*M*_w_), number-average molecular weight (*M*_*n*_), and the distribution (*M*_w_/*M*_*n*_) of the polymers were measured on a Waters GPC system, which was equipped with a Waters 1515 HPLC solvent pump, a Waters 2414 refractive index detector, and two Waters Styragel high resolution columns, at 40°C using HPLC grade THF as eluent at a flow rate of 0.35 mL/min. Monodispersed polystyrenes were used to generate the calibration curve. Absorption spectra were measured using a JASCO V-550 UV/VIS spectrophotometer. Fluorescence spectra were measured using a JASCO FCT-133 spectrometer.

### 2.4. Preparation of Polymer/siRNA Complex

The polymer was dissolved in methanol and diluted with RNase-free water to make solutions of different concentrations (10 to 0.1 *μ*M). The complexes were formed by gently mixing the siRNA solution with the polymer solution in equal volume and incubated for 30 min at room temperature. The final percentage of methanol in aqueous solution was below 5%.

The particle size and zeta potential were determined by a zeta-potential and particles size analyzer (ELSZ-2PL, Otsuka Electronics Co. Ltd., Tokyo, Japan).

### 2.5. Gel Retardation Assay for siRNA

To determine whether our polymers could retard siRNA migration, various ratios of polymer in complex with siRNA (1 *μ*M) were prepared with different molar ratios (the molar ratio of the cationic polymers to RNA) and incubated at room temperature for 30 min. Then the complex was mixed with loading buffer and applied to a 20% gel (Biocraft Co. Ltd., Tokyo, Japan), and electrophoresis was carried out for 60 min under a constant voltage of 100 V. The gel was stained with ethidium bromide for 20 min. After washing three times with water, the siRNA band was detected by a UV transmitter (ATTO Corporation, Tokyo, Japan). Two different loading buffers were used in this study. Loading buffer 1 contains glycerol (10%, v/v), 5 mM HEPES buffer (pH 7.3), and 1 mM EDTA. Loading buffer 2 has two more components: Triton X-100 (1%) and 60% of potassium polyvinyl sulfate (Wako Pure Chemical Industries, Ltd., Tokyo, Japan) to disrupt the polymer/siRNA complex.

### 2.6. Cellular Uptake by Monitoring the Fluorescence of Polythiophene

Next, 2 × 10^5^ cells (A549-luc) were cultured in a 12-well plate in F-12K with 200 *μ*g/mL geneticin. After 24 h, the polythiophene solution was added to the cell culture medium (final concentration was 0.5 *μ*M) and incubated for 72 h. The uptake was directly monitored by the fluorescence from polythiophene using a fluorescence microscope (Zeiss, LSM 510 Meta, Jena, Germany) and the intensity was measured by a Tali image-based cytometer (Life Technologies).

### 2.7. Cellular Localization of Polymers

Then, 2 × 10^5^ cells (A549-luc) were cultured on a glass bottom plate in F-12K with 200 *μ*g/mL geneticin. After 24 h, the 0.5 mM polythiophene solution was added to the cell culture medium (final concentration was 0.25 *μ*M) and incubated for 48 h. Confocal microscopy was performed on a Nikon Eclipse Ti-E inverted confocal fluorescence microscope (Nikon Instruments, Tokyo, Japan) using 60x oil immersion Plan Apo VC and 1.4-numerical aperture objective. Samples were excited with 488 and 561 nm solid-state lasers, and the emission was captured with a Nikon C2 confocal scan head (Nikon) interfaced to a PC running NIS-Elements C software. Three-dimensional stacks were generated from a series of confocal plane images with 1.0 *μ*m steps.

### 2.8. Knockdown Assay and Cytotoxicity Assessment

A549-luc cells were seeded at a density of 6 × 10^3^ cells/well in fresh F12-K medium containing 10% FBS without geneticin and incubated for 24 h. The polymer/siRNA complexes with different molar ratios were added to the culture medium, gently mixed, and then incubated for 72 h. As a positive control, cells were transfected with DharmaFECT1 transfection reagent (GE Healthcare, Lafayette, CO, USA)/siRNA complexes that were prepared according to the manufacturer's instructions. After 72 h, 100 *μ*L of the PicaGene LT2.0 luminescence reagent (Toyo Inki, Tokyo, Japan) was added to the cells and the luciferase activities were analyzed using a Multimode Plate Reader (EnSpire, PerkinElmer), according to the manufacturer's protocol. Cell viability was determined using the CellTiter-Glo kit (Promega, Madison, WI, USA), according to the manufacturer's protocol. The viability of nontreated control cells was arbitrarily defined as 100%.

## 3. Results and Discussion

### 3.1. Synthesis of PEI-*co*-polythiophenes Copolymers

Scheme 1 describes the synthetic route of the copolymers. Initially, polythiophenes** P1a** and** P2a** were obtained by oxidation coupling at monomer feed ratios of 50 : 50 and 75 : 25, respectively. We further grafted PEI onto the hydrophobic polythiophenes by reacting DSC activated hydroxyl groups on polythiophenes with the primary amines on PEI. The final copolymers were named** P1** and** P2** for convenience.

**Figure 1:**
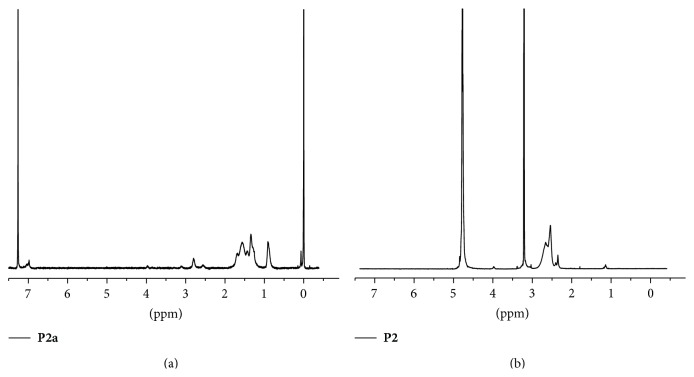
^1^H-NMR spectra of polythiophenes before (**P2a** in CDCl_3_) and after PEI conjugation (**P2** in* d*-methanol).

The ^1^H-NMR spectra in Figure 1 showed that the peaks from 0.9 to 1.7 ppm represent the protons on the alkyl side chain (–CH_3_ and –CH_2_–) of** P2a**. The weak signal at 7.0 ppm corresponds to the proton of the end-capped thiophene ring. The average molecular weights of** P1a** and** P2a** are 20 kg mol^−1^ (*M*_w_/*M*_*n*_ 1.85) and 32 kg mol^−1^ (*M*_w_/*M*_*n*_ 3.88), respectively (data not shown). After PEI conjugation to the side chain, the solubility of the copolymers significantly changed.** P1** and** P2** only dissolve in methanol and water and swell in chloroform. The ^1^H NMR spectrum of** P2** in* d*-methanol showed strong signals at ~2.5 ppm (Figure 1), which represents the protons of PEI-1.8K. Unfortunately, we did not obtain the GPC data of** P1** and** P2** because of poor solubility. Compared with a reported multistep approach involving a complicated fabrication of a conjugated polymer, the protocol presented herein is superior because it is simple and time effective.

### 3.2. UV-Vis and Fluorescence Spectra of Copolymers

**Scheme 2:**
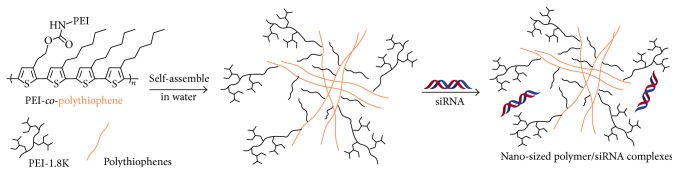
Demonstration of the self-assembly of copolymers in water and the formation of their nanosized complexes with siRNA.

**Figure 2:**
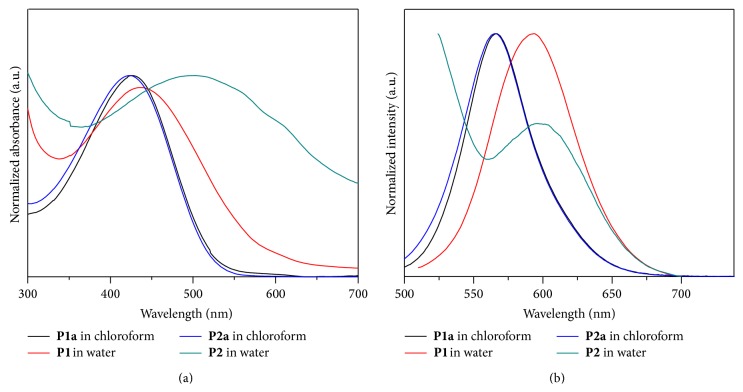
(a) UV-Vis absorption and (b) fluorescence spectra of copolymers in chloroform or in aqueous solution at room temperature. The value has been normalized.

**P1a** and** P2a** have absorption bands around 425 nm (Figure 2(a)), which are attributed to the coil conformation of polythiophenes in solution [23]. After conjugation with PEI, there is a slight red shift (436 nm) of the UV-absorption spectrum of** P1**. Surprisingly, with a lower PEI graft density (25%), the copolymer** P2** showed a broad absorption band at *λ* = 500 nm (Figure 2(a)), 70 nm higher than that observed for** P2a**. This is probably because of the formation of a self-assembly of** P2** in water displaying main chain aggregation. Charged PEI is expected to stretch as the hydrophilic shell, whereas the hydrophobic polyalkylthiophene functions as the core (Scheme 2). This process is associated with an aggregated chromophore backbone and a higher coplanarity. Therefore, this process leads to an increase in the conjugation length and a red shift of the UV-Vis absorption. As for the fluorescence properties, there is a similar wavelength maximum at 560 nm before PEI conjugation and at 590 nm for the PEI-conjugated graft copolymers (Figure 2(b)), which are easy to track by confocal microscopy at suitable excitation and emission ranges. It should be noted that the emission of** P2** was considerably less intense than that of** P2a**, presumably because of a fluorescence quenching by the aggregation of the conjugated main chains.

### 3.3. Formation of Polymer/siRNA Complexes

**Figure 3:**
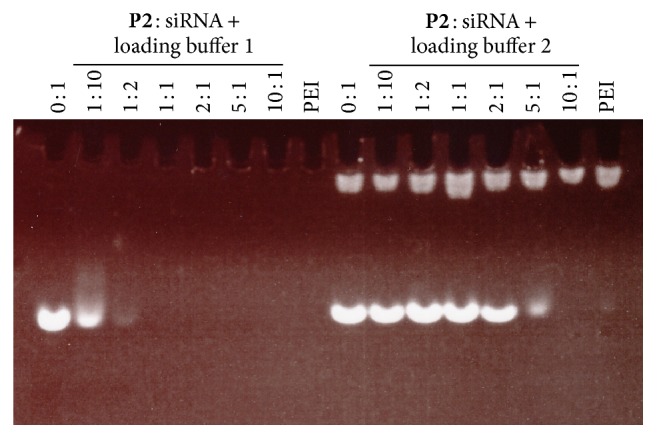
Copolymer** P2** forms complexes with siRNA at different molar ratios and the binding stability was tested by gel electrophoresis. Loading buffer 1 contains glycerol, HEPES buffer (pH 7.3), and EDTA. Loading buffer 2 has two more components: Triton X-100 (1% v/v) and potassium polyvinyl sulfate (PVSK) to disrupt the polymer/siRNA complex.

The binding capacity of siRNA with our cationic fluorescent copolymers was evaluated using a gel retardation assay at various molar ratios. As shown in Figure 3, the extent of retardation increases with the increasing ratio of** P2**/siRNA. By association with loading buffer 1, no obvious migrated siRNA bands were observed in the lanes where the molar ratio was larger than 1 : 2, indicating very strong binding by the** P2** polymer. Even with the disruption effect of loading buffer 2, which contains potassium polyvinyl sulfate to disrupt the polymer/siRNA complex, the migrating band became very weak at a 5 : 1 ratio and siRNA was still fully retarded by** P2** at a molar ratio of 10 : 1. For the** P1**/siRNA complex, the bands were slightly weaker at the same molar ratios (data not shown). Therefore, we assume that** P1** has slightly stronger siRNA binding capacity than** P2**, which is probably attributed to the higher PEI grafting density.

**Figure 4:**
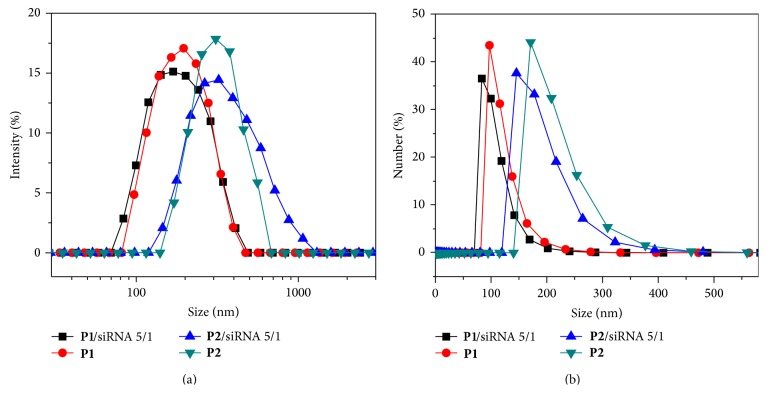
Particle size of polymer/siRNA complexes in water by DLS calculated by intensity (a) or number (b) average hydrodynamic diameters.

Although the siRNA complexes have a similar zeta potential of ~+30 mV, the complex sizes are quite different for the two polymers. As shown in Figure 4(a),** P1**/siRNA complexes were almost monodispersed nanoparticles with diameters of 184.9 ± 62.5 nm and a narrow polydispersity index (PDI, 0.121). In contrast, polymer** P2 **formed a larger size of complex with siRNA, with a broader PDI of 0.241 at the same molar ratio of 5 : 1. The lower molecular weight and better water solubility of** P1** may contribute to the smaller size and narrower distribution of their siRNA complexes. The diameter of the complex is a little smaller than the pure polymer nanoparticles in water. For example, the average sizes of** P1** and** P2** nanoparticles were determined to be 117.1 ± 26.3 and 208.0 ± 46.7 nm, which decreased to 104.1 ± 24.8 and 184.2 ± 46.3 nm after binding with siRNA at a 5 : 1 ratio (Figure 4(b)). This may be attributed to the slight condensation of ionized PEI by the negatively charged siRNA (Scheme 2). We investigated the cellular uptake and distribution of the fluorescent copolymer nanoparticles in the next step.

### 3.4. Cellular Uptake and Localization of Polythiophenes

**Figure 5:**
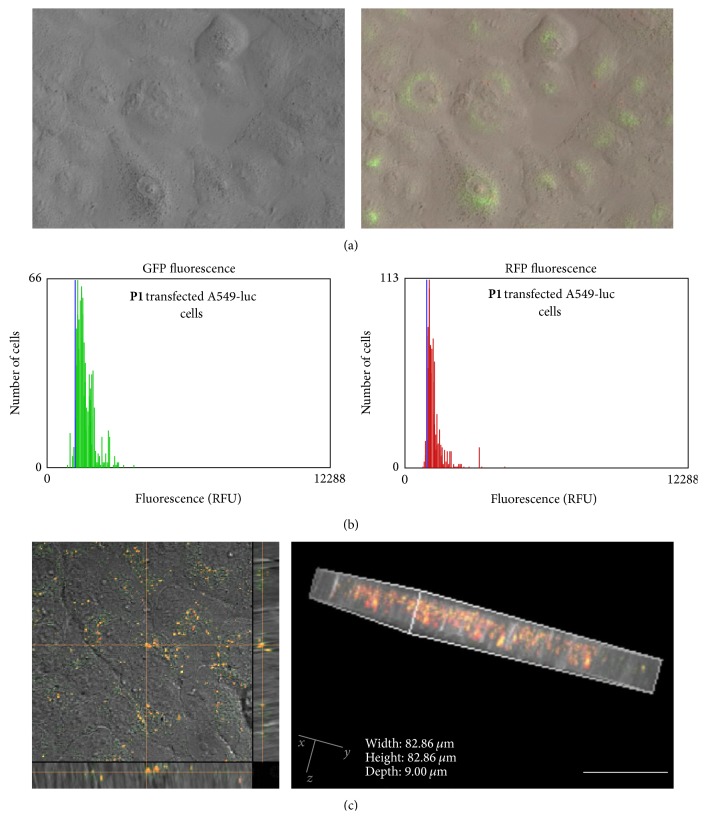
Copolymer** P1** (0.5 *μ*M) was transfected to the A549-luc cells for 72 h. (a) Fluorescence was detected by a fluorescent microscope, (b) determination of cellular uptake by a Tali image-based cytometer, (c) monitoring the localization of** P1** by three-dimensional stacks generated from a series of confocal plane images with 1.0 *μ*m steps.

Initially, the cellular uptake efficiency of polythiophenes was analyzed using fluorescence microscopy and Tali image-based cytometry. As shown in Figure 5(a), clear green and weak red fluorescence from** P1** were observed in the cytoplasm, but not in the nucleus of the cells, suggesting that uptake of** P1** by the cells was successful. By using an image-based cytometer, the uptake efficiency of** P1** was 81% (by green fluorescence at 466 nm) or 63% (by red fluorescence at 543 nm) (Figure 5(b)). Similar cellular uptake (~88%) was observed using the** P2**/Alexa647-labeled siRNA complex (data not shown), indicating good cell-penetrating activity of polythiophene-based copolymers.

Next, to identify the detailed location of polythiophenes in the delivery process,** P1** was added to the A549 cells and localization was determined by three-dimensional stacks generated from a series of confocal plane images with 1.0 *μ*m steps. According to Figure 5(c), a robust orange fluorescence was detected with a well-uniformed distribution in the cytoplasm. This indicated that** P1** was able to internalize into cells and escape endosomes to localize in the cytosol. These results clearly proved that our copolymers are very promising agents as intracellular delivery systems.

### 3.5. *In Vitro* Knockdown and Cytotoxicity Evaluation

**Figure 6:**
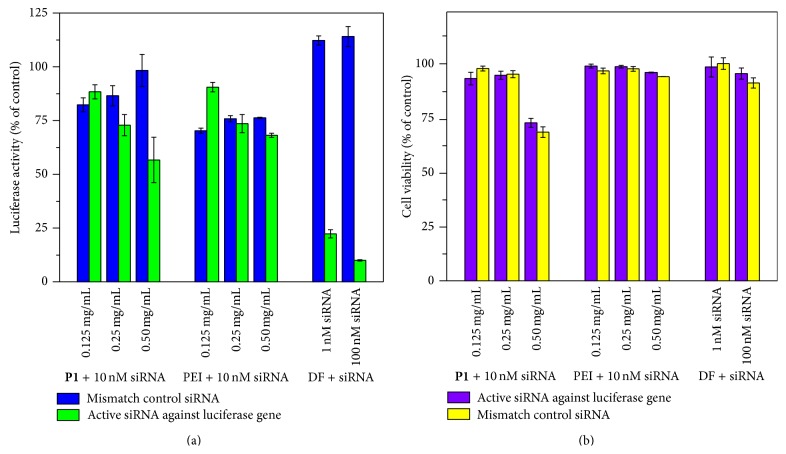
*In vitro* gene knockdown and cytotoxicity results of** P1**/siRNA and PEI-1.8K/siRNA complexes in A549-luc cells. (a) Relative expression of luciferase after 72 h incubation. (b) Cell viability determined using the CellTiter-Glo kit. The concentration of siRNA was fixed at 10 nM and DharmaFECT1 (DF) was used as a positive control carrier.

The goal of siRNA-based therapy is to knockdown the expression of a specific protein to achieve a specific therapeutic effect. Therefore, finally in this study, the luciferase gene-specific siRNA was chosen to evaluate the gene knockdown in A549-luc cells. DharmaFECT1, an efficient commercial transfection reagent, was used as positive control and showed the best luciferase knockdown efficiency (~80%). As for plain PEI polymer, there was very weak knockdown activity (<8.1%) and no obvious cytotoxicity when siRNA was applied with PEI-1.8K at different weight ratios (Figure 6(a)). This result corroborates with the previous report by Tian et al. and Yang et al. that low-molecular-weight PEIs have better biocompatibility but poor efficiency [10, 11]. After hydrophobic modification by** P1a**, the percentage knockdown of the luciferase gene was enhanced up to 41.6% at the 0.50 mg/mL** P1** complex with 10 nM siRNA. The corresponding cell viability of A549-luc was 72.3%. At a mid-dose of 0.25 mg/mL, the** P1**/siRNA complex showed 13.7% KD efficiency with cell viability of 94.8% (Figure 6(b)).

**Figure 7:**
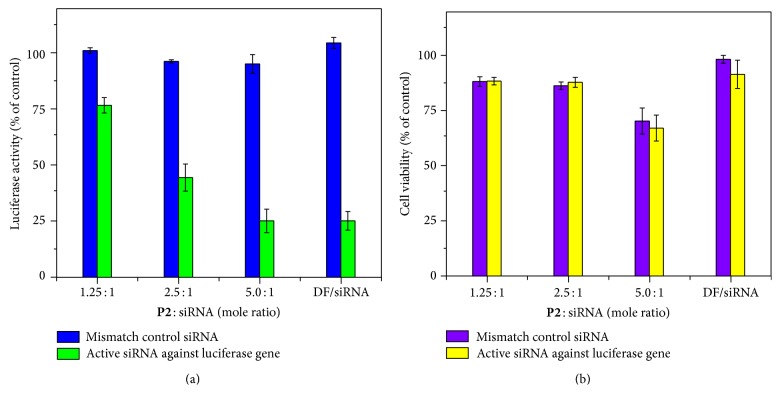
*In vitro* gene knockdown and cytotoxicity results of** P2**/siRNA complexes in A549-luc cells. (a) Relative expression of luciferase after 72 h incubation. (b) Cell viability determined using the CellTiter-Glo kit. The concentration of siRNA is fixed at 10 nM and DharmaFECT1 (DF) was used as a positive control carrier.

As for** P2** with more hydrophobic alkyl chain content, up to 52.8% luciferase knockdown was achieved with a single** P2**/siRNA treatment without obvious cytotoxicity (88% cell viability, Figures 7(a) and 7(b)), indicating protein-specific KD activity, but not because of the cytotoxicity of the materials. At a high molar ratio of 5 : 1, the** P1**/siRNA complex demonstrated a very high KD of 72.3%; however, the cell viability decreased to 68.6%. These results confirmed the effectiveness of our PEI-*co*-polythiophenes for gene delivery.

As gene silencing takes place at the mRNA stage in the cytosol, the fate of the DDS inside cells and intracellular localization must ensure the availability of siRNA in this compartment. In our design, PEI-1.8K should be the active compound to escape the endosomes. However, with the same RNA-binding capacity and surface positive charge, the** P1**/siRNA complexes with higher PEI density and smaller particle size showed much lower KD efficiency than hydrophobic** P2**. This implies that the hydrophobicity of the alkyl side chain also plays an important role in the effective delivery of siRNA.

We hypothesize here two possible factors that may explain the high uptake and good localization of our polymeric gene vectors. The first factor involves binding of the hydrophobic alkyl chain to the inner lipid bilayers of the cell membrane, thereby contributing to the high cellular uptake. The other factor is that the rigid polythiophene backbone may help the compound to penetrate the cell and other membranes, thus promoting internalization of DDS and also endosome escape. These two factors may define the higher performance of** P2** compared to the more hydrophilic** P1**. Further studies are required to clarify whether these two factors are responsible for the high uptake and knockdown efficiency. Finally, because siRNA delivery is an intricate and complicated multiple-step process, the effective tracking of our DDS is important for providing feedback to the optimization of carrier design and delivery efficiency [24].

## 4. Conclusions

Cationic and fluorescent copolymers based on PEI and polythiophenes have been designed and synthesized for siRNA delivery. The conjugated polythiophene endows the copolymer, as the DDS, with a label-free advantage, which was found to show good distribution inside A549 cells. The low-molecular-weight PEI-1.8K allows the graft copolymer to form nanosized stable complexes with siRNA with low cytotoxicity. Significant knockdown of the targeted protein expression was achieved by siRNA delivered through both** P1** and** P2** copolymers at an appropriate dosage. The hydrophobicity of both the thiophene main chain and the alkyl side chain may also contribute to the cellular uptake and drug delivery performance. In conclusion, this study demonstrated that PEI-*co*-polythiophenes copolymers might serve as novel, efficient, low toxic, and self-tracking siRNAdelivery vectors. We will focus on the function of hydrophobic interactions and the possible endosome escape mechanism of the fluorescent copolymers for gene delivery in future work.
